# Oncolytic Alphaviruses in Cancer Immunotherapy

**DOI:** 10.3390/vaccines5020009

**Published:** 2017-04-12

**Authors:** Kenneth Lundstrom

**Affiliations:** PanTherapeutics, CH1095 Lutry, Switzerland; lundstromkenneth@gmail.com; Tel.: +41-79-776-6351

**Keywords:** oncolytic alphaviruses, cancer immunotherapy, tumor eradication

## Abstract

Oncolytic viruses show specific targeting and killing of tumor cells and therefore provide attractive assets for cancer immunotherapy. In parallel to oncolytic viral vectors based on adenoviruses and herpes simplex viruses, oncolytic RNA viruses and particularly alphaviruses have been evaluated as delivery vehicles. Immunization studies in experimental rodent models for various cancers including glioblastoma, hematologic, hepatocellular, colon, cervix, and lung cancer as well as melanoma have been conducted with naturally occurring oncolytic alphavirus strains such as M1 and Sindbis AR339. Moreover, animals were vaccinated with engineered oncolytic replication-deficient and -competent Semliki Forest virus, Sindbis virus and Venezuelan equine encephalitis virus vectors expressing various antigens. Vaccinations elicited strong antibody responses and resulted in tumor growth inhibition, tumor regression and even complete tumor eradication. Vaccination also led to prolonged survival in several animal models. Furthermore, preclinical evaluation demonstrated both prophylactic and therapeutic efficacy of oncolytic alphavirus administration. Clinical trials in humans have mainly been limited to safety studies so far.

## 1. Introduction

Alphaviruses belong to the *Togaviridae* family and the Old World species such as Semliki Forest virus (SFV) and Sindbis virus (SIN) have been isolated in Africa, Asia, Australia and Europe [1]. On the other hand, the New World alphaviruses Venezuelan equine encephalitis virus (VEE), Eastern equine encephalitis virus (EEE) and Western equine encephalitis virus (WEE) originated in North and South America [2]. Alphaviruses have been associated with several epidemics in domestic animals and in humans worldwide [3,4,5]. For instance, EEE and WEE have been reported as causing severe neurological diseases and fatalities in horses and humans with symptoms ranging from fever to encephalomyelitis, with a mortality of 90% [6]. Lifelong immunity against alphaviruses was discovered in survivors. Similarly, SFV and SIN have been associated with several epidemics in Africa [7,8] and acute VEE infections have been responsible for the death of two individuals in the Amazon region of Peru [9]. Chikungunya virus (CHIK) has been determined responsible for recent outbreaks in the Republic of Congo [10], the island of Reunion [11] and the Bahia state in Brazil [12].

**Figure 1:**
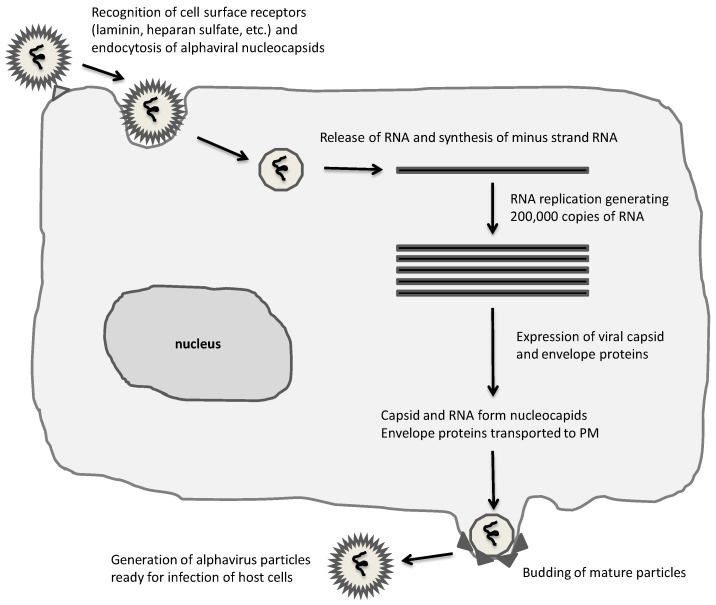
Alphavirus life-cycle. A broad range of mammalian cells are susceptible to alphaviruses, which are taken up by endocytosis and RNA released for immediate replication in the cytoplasm. Extensive RNA replication and expression of capsid and envelope proteins result in assembly and budding of mature infectious alphaviral particles.

The broad host range in both invertebrates (mosquitoes and hematophagous insects) [13] and vertebrates (mammals, birds, amphibians, reptiles and fish) [3] is characteristic of alphaviruses. However, the life cycle shows significant differences in arthropod hosts compared to vertebrates [1] (Figure 1), where a persistent lifelong infection with hardly any cytotoxicity is observed in arthropods. In contrast, apoptosis and rapid death occur in vertebrate cells. There has been much speculation about host cell receptors recognizing alphaviruses, which include MHC HLA-A and HLA-B in humans and H-2K and H-2D in mice [14], laminin receptors [15] and heparan sulfate receptors [16]. After receptor recognition and attachment to the cell surface, the alphavirus envelope fuses with the host plasma membrane, the viral particle enters the cell through endocytosis and nucleocapsids are released [17,18] followed by highly efficient RNA replication in the cytoplasm by the RNA replicase complex consisting of the nonstructural proteins 1-4 (nsP1-4) [1]. At approximately three hours post-infection, host cell protein synthesis is taken over by viral capsid and envelope protein expression [19] and nucleocapsids are assembled and transported to the plasma membrane. The envelope proteins traverse the endoplasmic reticulum (ER) and the Golgi complex for the assembly of mature viral particles on the plasma membrane [20,21] and release of virions by budding [22].

Application of alphavirus-based vectors for immunization studies has understandably raised safety concerns. To address this issue, vector engineering has mainly been based on attenuated laboratory strains, which are not considered to be pathogenic [23]. Moreover, replication-deficient viruses have been favored in many vaccine studies [24]. A number of cancer vaccine and immunotherapy studies have been conducted with alphavirus vectors [25]. In this context, SFV, SIN and VEE vectors have been administered in the form of recombinant particles, RNA replicons and layered DNA vectors [26,27]. Tumor protection was obtained in mice immunized with SFV and VEE particles expressing the *P1A* gene [28] and the human papilloma virus (HPV) *E7* gene [29,30], respectively. Immunization with SFV vectors expressing a type 16 HPV E6–E7 fusion protein provided both prophylactic [30] and therapeutic [31] responses in mice. Furthermore, intramuscular administration of only 1-µg SFV RNA replicons carrying the *LacZ* gene provided complete protection for mice challenged with tumor cells and extended the survival by 10–20 days in animals with pre-existing tumors [32]. Layered SIN-neu DNA vectors were applied for immunization of mice with implanted breast tumors, which resulted in significant tumor regression [33]. In attempts to further improve immunogenicity, the pSFV-10E vector providing 10-fold increase in expression levels was applied for intratumoral administration of SFV-IL-12 (interleukin-12) particles in BALB/c mice bearing K-BALB tumors, which resulted in complete tumor regression [34]. Similarly, treatment of CT26 tumors and 4T1 metastatic tumors demonstrated efficient tumor growth inhibition. Furthermore, expression of IL-18 from the pSFV-10E vector resulted in tumor regression in subcutaneous K-BALB and CT26 tumors in BALB/c mice [35]. Significant inhibition of tumor growth and metastatic spread in CT26 colon carcinoma and 4T1-metastasizing mammary carcinoma models was observed in mice after prophylactic or therapeutic immunization of mice with SFV particles expressing vascular epidermal growth factor receptor-2 (VEGFR-2) [36]. In addition to acquired protection in animal models, alphavirus particles have been subjected to clinical trials. For instance, the carcinoembryonic antigen (CEA) in colorectal cancer patients [37] and prostate-specific membrane antigen (PSMA) in patients with metastatic cancers [38], respectively, demonstrated good safety profiles although the immunogenicity responses have been modest.

In attempts to further improve the immune responses, one approach has been to employ oncolytic viruses. Oncolytic viruses preferentially target and kill cancer cells by presenting a cytolytic effect and contributing to antigen release and induction of anti-cancer adaptive immunity, which supports the elimination of distant metastases and long-term anti-cancer immune surveillance. Both naturally occurring and engineered oncolytic alphaviruses, which exploit cancer-specific changes in cellular signaling aiming at targeting cancers and their microenvironment have been applied and evaluated for cancer immunotherapy [39]. In this review, oncolytic alphaviruses are described and examples of their application in prophylactic and therapeutic settings are presented.

## 2. Expression Vectors for Alphaviruses

**Figure 2:**
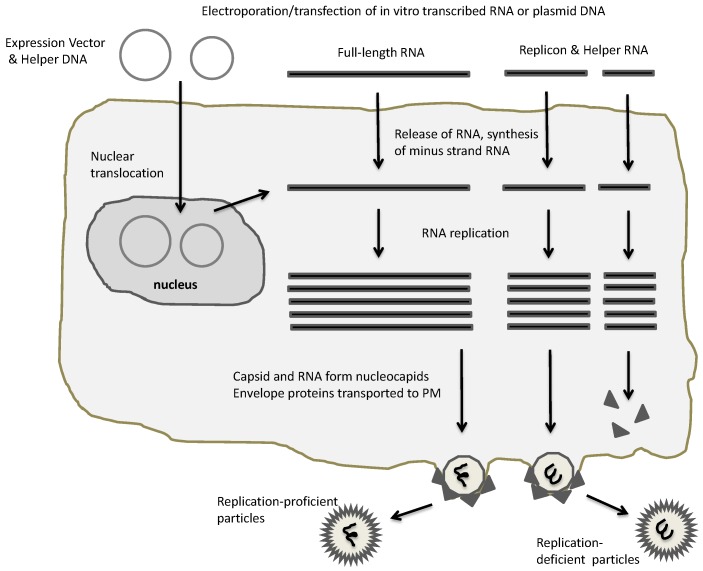
Alphaviral expression systems. Electroporation or transfection of plasmid-based alphaviral DNA vectors or in vitro transcribed RNA generates RNA replication and packaging of recombinant alphaviral particles. In case of full-length RNA, replication-proficient particles are generated, whereas application of replicon and helper RNA results in replication-deficient particles. PM, plasma membrane.

**Figure 3:**
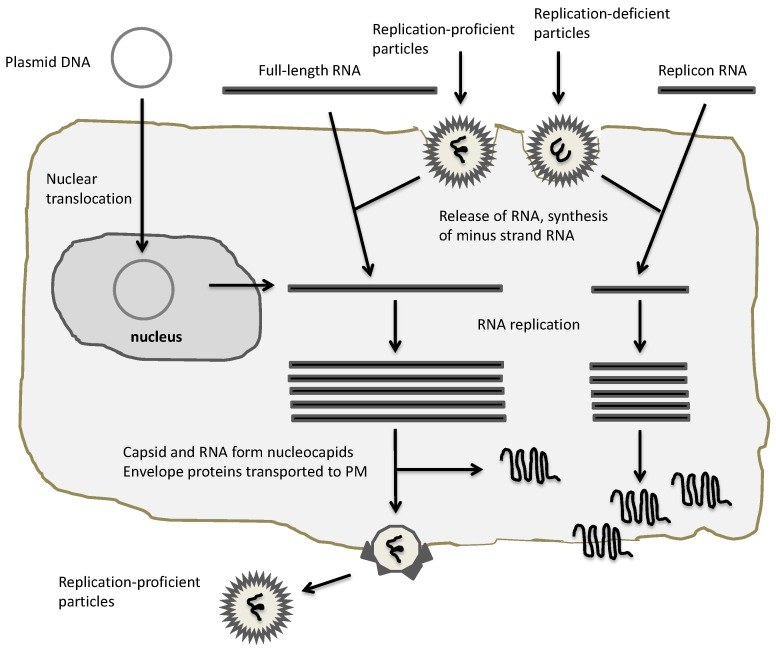
Application of alphaviral expression vectors. Plasmid DNA/RNA replicons and recombinant particles can be used for heterologous expression. In the case of DNA and RNA replicon delivery, appropriate transfection methods for host cells have to be established, whereas recombinant particles naturally provide a broad range of host cell susceptibility. All vectors will provide expression of heterologous proteins, but only full-length RNA and replication-proficient vectors will provide new virus progeny.

Three main types of expression vector systems have been engineered for SFV [40], SIN [41] and VEE [42] (Figure 2) referred to as replication-deficient, replication-proficient and layered DNA vectors [25]. In all cases, the replication complex consisting of the nsP1-4 proteins is employed for highly efficient RNA replication, which combined with utilization of the strong 26S alphavirus subgenomic promoter generates high level transgene expression. Administration of in vitro transcribed RNA from the alphavirus expression vector carrying the alphaviral non-structural genes and the gene of interest can be directly used for transfection of naked replicon RNA [40] (Figure 3). Co-transfection of in vitro transcribed RNA from the alphavirus expression vector with alphavirus helper vector containing the viral structural genes produces replication-deficient recombinant particles. Transfection or infection of host cells with in vitro transcribed full-length alphavirus RNA or particles, respectively, generates replication-proficient particles. The replacement of the SP6 promoter with the RNA polymerase II-dependent cytomegalovirus early (CMV 1E) enhancer/promoter generated layered DNA SFV [43] and SIN [44] vectors, which can be applied for transfection of host cells.

Significant efforts have been dedicated to the engineering of alphavirus vectors in attempts to enhance transgene production and prolong the duration of gene expression. Introduction of mutations into the non-structural genes of SFV [45,46] and SIN [47,48] has generated reduced cytotoxicity of host cells, also providing extended expression time at higher levels. For instance, engineering of a single point mutation in the SIN *nsP2* gene resulted in persistent infection in transduced mammalian cells [49]. In the case of SFV, vector development has also included avirulent strains showing reduced cytoxicity in vivo. In this context, the *nsP1-4* genes from the avirulent SFV A7(74) strain were introduced into an SFV expression vector, which when used in the form of replication-deficient particles showed reduced cytotoxicity and temperature-dependent expression in mammalian cell lines and primary neurons [50]. In another approach, the translation enhancement signal (TES) from the SFV capsid protein was introduced into the SFV expression, which generated 5–10-fold increase in heterologous gene expression levels [51]. To eliminate any unfavorable function of the produced fusion protein, the foot-and-mouth disease virus 2A (FMDV 2A) ribosome-skipping sequence was introduced downstream of the capsid TES sequence to provide an authentic protein structure [52]. VEE self-replicating subgenomic RNA replicons have also been designed to address the question of underused VEE replicon enzymes, which resulted in 10–50-fold increase in protein expression [53]. Although alphavirus particles can accommodate reasonably large foreign RNA sequences, introduction of the Ser 180/Gly183 point mutations into the SIN capsid protein generated significantly larger viral particles (205 nm in comparison to the normal size of 70 nm), which allowed packaging of up to 18 kb RNA without any significant titer reduction [54].

Uncontrolled propagation of replication-competent alphavirus particles through recombination events between expression and helper vectors poses safety concerns, which has triggered engineering of modified helper vectors. In this context, the second-generation SFV-Helper2 vector was generated by introduction of three point mutations in the p62 precursor to prevent its cleavage into E2 and E3 envelope proteins [55]. SFV particles generated with the SFV-Helper2 vectors are conditionally infectious, therefore requiring activation by α-chymotrypsin. This approach significantly reduced the rate of homologous recombination and production of replication-competent particles. To further increase safety, recombination events between alphavirus expression and helper vectors have been eliminated by the engineering of split helper vectors for both SFV [56] and SIN [57], in which the capsid and envelope genes are located on separate helper vectors. In another approach, propagation of replication-proficient SIN particles was controlled by fusion of the Herpes simplex virus type-1 *thymidine kinase* (*HSV-TK*) gene with the *nsP3* subunit of the viral replicase [58]. Administration of the prodrug ganciclovir (GCV) demonstrated efficient shut off of virus propagation in vivo.

## 3. Oncolytic Alphavirus Approaches

The need for improved targeting and efficacy of viral delivery for cancer therapy has encouraged the evaluation of oncolytic viruses (Table 1). Several approaches have been tested to provide tumor targeting of alphaviruses. A classic application comprises introduction of immunoglobin G (IgG) binding domains of protein A into the SIN *E2* envelope gene, which resulted in 10^5^-fold reduced infection of BHK-21 cells and simultaneously enhanced infection of cells treated with a monoclonal antibody against surface proteins [59]. However, although this approach showed some promise in cell lines, it has never been reported for tumor targeting in animal studies. Surprisingly, natural tumor targeting was observed for SIN-Luc particles in vivo, showing an increase in luciferase concentration in tumor xenografts [60]. Therapeutic efficacy was obtained after subcutaneous administration of SIN-IL-12 particles. Further tumor targeting was demonstrated by intraperitoneal injection of SIN-Luc particles, which resulted in efficient delivery to fibrosarcomas implanted in the mouse tail. Moreover, the tumor-targeting oncolytic SIN vector expressing tumor-associated antigens (TAAs) demonstrated enhanced therapeutic effect in mice bearing subcutaneous, intraperitoneal and lung cancers [61]. However, in this case the SIN-TAA efficacy was not dependent on tumor cell targeting, but due to transient expression of TAAs in lymph nodes draining the injection site. Based on the findings of natural tumor targeting of SIN particles, biodistribution of SFV particles was evaluated after intravenous, intraperitoneal and intratumoral administration. However, no tumor targeting of luciferase expression was observed, suggesting that the natural targeting observed for SIN was not a common feature for alphaviruses [62]. Moreover, related to the SIN tropism, in contrast to earlier findings suggesting laminin receptors as targets for SIN, it was discovered that although JB leukemia cells express high levels of laminin receptors, they showed low susceptibility to SIN [63]. In another study, tumor tropism was investigated in a human ML-14a xenograft model, which indicated that SIN infections are not defined by the level of SIN receptors, but by interferon (IFN) response in tumors [64]. High susceptibility for SIN was observed in those cells that showed defects in either IFN production or signaling. 

The replication-competent SIN AR339 strain was demonstrated to induce cytopathic effects and apoptosis in HeLaS3 and C33A cervical cancer cells and in HOC-1, HAC-2 and OMC-3 ovarian cancer cells, but not in normal keratinocytes in vitro [65]. Furthermore, significant regression of established cervical tumors was observed after intratumoral and intravenous administration of SIN particles in nude mice. A single systemic injection of SIN also induced necrosis within tumors at remote sites and in the metastasis model of ovarian cancer suppression of ascites formation was seen. Moreover, in vivo green fluorescent protein (GFP) imaging confirmed specific SIN tumor targeting. The oncolytic SIN AR399 strain has been analyzed for cytotoxicity and growth in 13 oral squamous cell carcinoma (OSCC) cell lines [66]. SIN induced cytopathic effects in all OSCC cell lines, except in HSC-4 cells. The cytotoxicity was comparable to reovirus infections and resulted in apoptotic cell death. This suggests that naturally occurring oncolytic SIN vectors can be attractive for cancer therapy [67]. Moreover, the oncolytic SINV AR339 strain was evaluated for cytotoxicity and viral growth in five human neuroblastoma cell lines (SK-N-SH, IMR-32, LAN-5, GOTO and RT-BM-1) and in vivo in nude mice implanted with neuroblastoma xenografts [68]. SIN AR339 induced significant cytotoxicity and mRNA levels of the anti-apoptotic genes *Bcl-2* and *Bcl-xL* were increased in LAN-5 and RT-BM-1 cells. Intratumoral and intravenous administration caused substantial neuroblastoma regression in nude mice. In another application, oncolytic SIN vectors expressing HSV-TK ensured sufficient prodrug GCV conversion and activation for bystander effect, killing surrounding untransduced tumor cells [69]. An advantage of this approach is that the HSV-TK activity in tumors can be non-invasively monitored by positron emission tomography (PET).

The oncolytic potential of the replication-proficient SFV VA7-EGFP virus, based on the avirulent SFV A7(74) strain, was evaluated in tumor cell lines and in vivo [70]. Most of the studied cancer cell lines were infected by SFV VA7-EGFP, leading to complete cell lysis within 72 h. A single intraperitoneal, intravenous or intratumoral injection of 10^6^ plaque forming units (pfu) of virus generated a significant regression of grafted human A2058 melanoma tumors in severe combined immunodeficiency (SCID) mice. It was of some concern that SFV particles had spread to the brain (according to histological analysis), resulting in neuropathology. The SFV VA7-EGFP virus was evaluated in another study in immunodeficient mice and immunocompetent rats [71]. Intratumoral administration to NMRI nu/nu mice implanted with subcutaneous A549 human lung adenocarcinoma inhibited almost completely tumor growth, while intravenous injection only delayed it. Intraperitoneal administration showed no response. The lack of response reflected at least partly the strong type I IFN response occurring in tumors. Although the immunized animals showed no abnormal behavior, SFV-positive foci were detected in the brain. Intracranial injection of SFV VA7-EGFP in rats carrying BT4C glioma resulted in significant tumor reduction. However, increase in tumor size occurred again after a lag period and none of the treated rats survived. Furthermore, SFV VA7-EGFP has been evaluated in vitro in U87, U251 and A172 human glioma cells and in vivo in subcutaneous and orthotopic tumor models in BALB/c mice [72]. Efficient killing of the three glioma cell lines was observed and intravenous administration of SFV VA7-EGFP completely eradicated 100% of small and 50% of large subcutaneously implanted U87Fluc tumors. Moreover, long-term survival was achieved in 16 of 17 mice. It was also demonstrated that SFV was well tolerated and it caused no damage to heart, liver, spleen and brain. Similarly, SIN vectors expressing GFP showed preference for U87MG glioblastoma cells, strong cytolytic action and selective killing of human glioblastomas in vivo [73]. Furthermore, a local isolate of CHIK demonstrated efficient infection of the U87MG glioblastoma cell line, resulting in apoptosis 48 h post-infection, suggesting the potential use of oncolytic CHIK for treatment of glioblastoma [74].

In another study, locally administered SFV VA7-EGFP increased the survival rate in mice with implanted A549 lung adenocarcinoma cells [75]. However, the systemic delivery did not elicit significant anti-tumor responses, which might be related to the route of administration, immunological barriers and IFN responsiveness. Furthermore, SFV VA7-EGFP was compared to the commonly used oncolytic adenovirus Ad5Delta24 [76]. SFV VA7-EGFP showed faster oncolysis and higher efficiency at low multiplicity of infection than the adenovirus vector in vitro. Tumor size was reduced in subcutaneous human osteosarcoma xenografts in nude mice similarly after SFV and adenovirus administration. For SFV VA7-EGFP treatment the tumor regression was either complete or reduced to pinpoint size. When SFV-VA7-EGFP was administered in orthotopic osteosarcoma nude mice with highly aggressive tumor growth, the survival was significantly extended although no complete cure was achieved.

Relating to brain tumors, SFV expressing IL-12 was evaluated in a syngeneic RG2 rat glioma model [77]. Low dose (5 × 10^7^ particles) treatment resulted in 70% reduction in tumor volume and significantly extended survival, while the high dose (5 × 10^8^ particles) generated an 87% decrease in tumor volume. The observed effect was related to the oncolytic activity of the virus and the SFV-based IL-12 expression. Treatment-related death due to inflammation, necrosis and edema was described after high dose treatment. In another study, attempts to address the restricted replication of SFV in malignant gliomas comprised combination treatment with vaccinia virus (VV) [78]. Unexpectedly, both SFV and VV were unable to infect parenchymal brain tumors in mice. These findings triggered the investigation on clonal variation on interferon responses [79]. Two SFV VA7-sensitive clones of CT26 mouse colon carcinoma were evaluated for tumor regression. Both the CT26WT and the CT26LacZ clones secreted active IFN in vitro after SFV infections, but only CT26WT cells were protected. Transcriptome sequencing and protein expression analyses revealed that 56 different genes associated with pattern recognition and IFN-1 signaling pathways were constitutively expressed in CT26WT cells. CT26WT tumors were SFV-resistant in vivo, whereas for CT26LacZ tumors complete eradication was obtained both in immunocompetent and SCID mice. When mice were subjected to SFV administration of double-flank transplantations, CT26WT tumors continued to grow while CT26LacZ tumors located on the contralateral flank were eradicated. The favorable findings of tumor regression in rodent tumor models encouraged studies on SFV VA7-EGFP in the canine osteosarcoma cell lines Abrams and D17 and in vivo in two adult dogs (Beagles) [80]. SFV VA7-EGFP was demonstrated to replicate in Abrams and D17 cells, killing them. Furthermore, a single intravenous injection of 2 × 10^5^ pfu of SFV VA7-EGFP produced no adverse events in Beagles and no infectious virus could be recovered from collected samples.

In addition to targeting solid tumors, SIN vectors were demonstrated to efficiently trigger apoptosis in murine BW5147 malignant hematopoietic T-cells [63]. In contrast, only modest efficacy was seen in human lymphoma and leukemia cells. For in vivo evaluation, BW5147 cells were intraperitoneally administered into (C3HXAKR) F1 hybrid mice, which revealed SIN targeting of tumors and a significantly prolonged survival of tumor-bearing animals.

Due to some limitations of repeated injections, a strategy of employing SFV and VV vectors for the first and second immunizations, respectively, or vice versa has been evaluated [81]. Enhanced anti-tumor effects were obtained in tumor-bearing mice when the vector was switched from SFV to VV after the first immunization. Likewise, murine ovarian surface epithelial cancer (MOSEC) tumors showed increased regression after VV immunizations followed by SFV administration. Furthermore, immunization with VV-ovalbumin (OVA) and SFV-OVA particles resulted in enhanced OVA-specific CD8^+^ T-cell immune responses. The in vitro tumor killing and in vivo anti-tumor effects were due to a combination of viral oncolysis and antigen-specific immunity.

The synergistic effect of oncolytic alpahvirus vectors and checkpoint inhibitors was achieved by applying a non-replicative SFV vector expressing IL-12 and an anti-PD1 monoclonal antibody (mAb) to induce tumor regression and prolonged survival [82]. SFV-IL12 particles combined with anti-PD1 mAb were intratumorally administered in mice with implanted MC38 colon and B16-OVA melanoma tumors. Only a marginal increase in cytotoxic T-lymphocyte (CTL) response was obtained for the combination therapy. However, as SFV-IL12 induced PD-L1 expression in an IFN-γ-dependent fashion in tumor cells, the PD-L1 mediated adaptive resistance could be addressed by the combination therapy.

An interesting recent finding was the identification of the naturally occurring oncolytic alphavirus M1, discovered to selectively kill zinc-finger antiviral protein (ZAP)-deficient cancer cells [83]. M1 demonstrated potent oncolytic efficacy and high tumor tropism in vitro, ex vivo and in vitro. The selective dependency on ZAP deficiency was confirmed by a large-scale multicenter pathology study based on tissue microarrays. It was also discovered that M1 induced endoplasmic reticulum (ER) stress-mediated apoptosis, which contributed to cancer cell death. It has been further demonstrated that activation of cyclic adenosine monophosphate (cAMP) significantly sensitized refractory cancer cells to M1 in vitro, ex vivo and in vivo [84]. Moreover, activation of the cAMP signaling pathway inhibited M1-induced expression of antiviral factors contributing to prolongation of ER stress and apoptosis. The safety of M1 was evaluated in cynomolgus macaques by six intravenous injections of 1 × 10^9^ pfu/dose [85]. A number of parameters such as body weight, temperature, complete blood count, clinical biochemistry, cytokine profiles, lymphocyte subsets, neutralizing antibodies, magnetic resonance imaging and clinical symptoms were monitored. The outcome showed no clinical, biochemical, immunological or medical imaging or other pathological evidence of toxicity, confirming the safety of systemic delivery of M1.

Oncolytic viruses have suffered from inefficient extravasation from tumor blood vessels due to their large particle size. To facilitate the delivery to cancer cells, the modulation of tumor vascular leakiness by vascular epithelial growth factor (VEGF) and/or metronomic chemotherapy, significantly enhanced tumor vascular permeability of SIN particles, providing enhanced tumor targeting [86]. Another approach to improve the safety and to target the replication to tumor cells only, has been to incorporate tissue-specific micro-RNAs (miRNAs) into alphavirus vectors [87]. In this context, six tandem targets for the neuron-specific miR124 sequences were introduced between the *nsP3* and *nsP4* genes in the SFV4 genome. The engineered SFV4-miRT124 particles showed glioma targeting and only a limited spread within the central nervous system after intraperitoneal injections in BALB/c mice. Moreover, the survival of animals was significantly prolonged. Strong protective immunity against SFV was achieved in vaccinated animals. Intracranial injections into adult mice demonstrated significantly increased resistance of neurons, while oligodendrocytes in the corpus callosum were susceptible to SFV4-miRT124. As IFN-1 generally strongly interferes with antiviral tumor responses, the effect of viral replication and oncolysis was evaluated for SFV-miRT124 [88]. It was demonstrated that SFV-miRT124 showed tolerance to IFN-1 and increased oncolytic potency in mouse CT-2A astrocytoma cells and in human glioblastoma cell lines pretreated with IFN-1. A single intraperitoneal injection of SFV-miRT124 demonstrated replication in CT-2A orthotopic gliomas implanted in C57BL/6 mice and generated significant inhibition of tumor growth and provided improved survival rates for the animals. This approach presents an opportunity to provide controlled replicative potency for improved oncolytic alphavirus vectors.

## 4. Comparison to Other Oncolytic RNA Viruses

More or less all viral vector systems including adenoviruses, adeno-associated viruses (AAV), herpes simplex viruses (HSV), lentiviruses, retroviruses, and vaccinia viruses have been utilized for cancer immunotherapy studies [24]. The outcome has been promising with strong immune responses and protection against challenges with lethal doses of tumor cells in various animal models. Further encouragement was obtained by the approval of the first viral drug based on HSV for melanoma treatment [89]. In the context of oncolytic RNA viruses, measles viruses (MV), and rhabdoviruses have been used in cancer immunotherapy in addition to alphaviruses.

The Edmonston-B (MV-Edm) is an attenuated MV strain, which can selectively infect and replicate in tumor cells demonstrating anti-tumor activity without causing significant cytotoxicity to normal tissue [90]. Evaluation of MV-Edm has been carried out in different cell lines, primary cancer cells and xenograft and syngeneic models such as B-cell non-Hodgkin lymphoma [91], ovarian cancer [92], glioblastoma multiforme [93], breast [94] and prostate [95] cancers. Intratumoral injection of MV-Edm induced regression of implanted human lymphoma xenografts in SCID mice [91]. Furthermore, combination therapy of MV expressing carcinoembryonic antigen (CEA) and thyroidial sodium iodide symporter (NIS) in mice with SKOV2ip.1 ovarian xenografts was superior compared to individual treatment with either MV-CEA or MV-NIS [92]. MV-CEA has also been verified in MDA-MB-231 mammary tumor [94] and subcutaneous prostate PC-3 xenograft [95] models, showing significant delay in tumor growth and prolonged survival. In another approach, the CD46 and the signaling lymphocytic activation molecule (SLAM) sequences have been incorporated into the hemagglutinin protein sequence with a single-chain antibody against epidermal growth factor receptor (EGFR) at the C-terminus in attempts to target tumors [93]. Intratumoral inoculation of the modified MV-Edm resulted in tumor regression and significantly extended survival. Currently, MV-CEA and MV-NIS viruses are subjected to clinical trials against ovarian cancer, glioblastoma multiforme, multiple myeloma, mesothelioma, head and neck cancer, breast cancer and malignant peripheral nerve sheath tumors [96].

The most prominent applications of rhabdoviruses relate to vesicular stomatitis virus (VSV). In this context, VSV matrix (M) protein mutants represent oncolytic viruses exhibiting low virulence in vivo. However, cancer cells maintain resistance to oncolytic VSV due to residual antiviral responses. To overcome this problem, combination therapy with natural agents such as curcumin, resveratrol and flavokavain B was evaluated [97]. Pretreatment with curcumin potentiated VSV-induced oncolysis in PC-3 prostate cancer cells and in vivo in a mouse prostate cancer model. Curcumin did not affect the expression of type I IFN, but it inhibited the phosphorylation and activation of signal transducer and activator of transcription 1 (STAT1), which is a key player in the IFN response pathway. Furthermore, tumor-selective oncolytic VSV showed in a MPC-11 mouse plasmocytoma model extensive intratumoral viral replication, sustained viremia, intravascular coagulation and a rapidly fatal tumor lysis syndrome (TLS) even at a low dose [98]. Moreover, oncolytic VSV has proven highly efficient in glioblastomas and more recently in ovarian cancer, where combination with the JAK1/2-inhibitor ruxolitinib provided superior efficacy [99]. In attempts to suppress the natural neurotoxicity of VSV vectors, chimeric pseudotyped vectors with retained oncolytic potency have been engineered. For instance, introduction of the lymphocytic choriomeningitis virus glycoprotein (LCMV-GP) into the envelope of replicating VSV particles, effectively eliminated brain cancer in several in vivo tumor models, while normal brain cells were unaffected [100]. Furthermore, VSV-LCMV-GP managed to escape humoral immunity and allowed repeated systemic vector administration. Similarly, chimeric VSV particles with a Lassa virus glycoprotein precursor (LASV-GPC) envelope showed no adverse effect on normal brain cells, but targeted and destroyed tumor cells including high-grade glioblastomas [101]. Intratumoral injection in one brain tumor of mice implanted with two brain tumors led not only to destruction of the targeted tumor, but resulted in selective infection of the second contralateral tumor. Moreover, intratumoral administration of the Maraba MG1 virus (belonging to the genus of *Vesiculovirus*) expressing IL-12, promoted the migration of active natural killer (NK) cells and contributed to the reduction of tumor burden and improved survival in a colon cancer model of peritoneal carcinomatosis [102].

In comparison to oncolytic alphaviruses, MV and rhabdoviruses have proven efficient in immunization studies and have demonstrated significant tumor regression in various tumor models. Interestingly, enhanced efficacy of VSV vectors has been obtained in combination therapy with natural agents previously described as therapeutics for cancer treatment. This approach should also be investigated for alphaviruses. Perhaps the biggest difference between the utilization of oncolytic MV and rhabdoviruses in cancer immunotherapy is their single-stranded RNA (ssRNA) genome of negative polarity in contrast to alphaviruses possessing a positive ssRNA genome. Although packaging systems have been engineered for negative ssRNA viruses, the straight forward high titer production of alphavirus particles is an asset in cancer immunotherapy. Moreover, the flexibility of utilization of recombinant particles, RNA replicons and layered DNA vectors are certainly of additional advantage.

## 5. Discussion

Since the beginning of cancer immunotherapy the goal has been to develop delivery methods for tumor-associated antigens, cytokines and other potential immunostimulatory molecules. Due to the limitations of direct administration of for instance interleukins, the possibility to employ viral and non-viral delivery systems has become attractive. Interestingly, a number of viral vectors have been evaluated in immunization studies in animal tumor models and even in clinical trials [24]. Among non-viral delivery vehicles the single-stranded aptamers provide an attractive alternative as they possess high specificity, selectivity and binding affinity towards their targets [103]. In this context, aptamers can be engineered to bind to such diverse targets as proteins, cells and tissues and should be seriously considered for cancer therapy. Steady progress in viral vector development and especially application of oncolytic viruses has also resulted in success [104]. A number of oncolytic adenovirus and HSV vectors have been subjected to preclinical studies and clinical trials. Particularly, an advanced-generation HSV vector optimized for oncolytic and immunomodulatory activities was after a phase III trial approved in the US and Europe for treatment of cutaneous and subcutaneous melanoma.

Oncolytic alphaviruses have seen a number of applications in rodent models for various types of cancers ranging from brain cancers to lymphomas, leukemia and melanomas (Table 1). Although some variation in response has been observed, inhibition of tumor growth and tumor regression has been achieved, further resulting in prolonged survival of immunized animals. In several cases, complete tumor eradication has been obtained and vaccination has provided protection against challenges with lethal doses of tumor cells. In comparison to conventional alphavirus vectors, the tumor-specificity of oncolytic vectors has provided additional efficacy. The natural tumor targeting of SIN [60] and the recent discovery of the naturally oncolytic M1 alphavirus [83] represent potential assets for enhanced cancer immunotherapy applications. Several studies have been conducted with the attenuated SFV VA7 vector [70,71,72,75,76] resulting in therapeutic effect in immunized mice. Clearly, a stronger impact was obtained after intratumoral injections than systemic administration. Certain concerns have also been raised by the potential spread to the brain of oncolytic alphaviruses [70]. In comparison to oncolytic adenovirus (Ad5Delta24) delivery, SFV VA7 showed faster oncolysis and higher efficiency of infection [77]. Another approach to engineer “oncolytic” vectors of conventional recombinant SFV particles has been to encapsulate SFV particles in liposomes, demonstrating passive targeting of tumors [105]. This approach allowed enhanced tumor-specific expression of β-galactosidase after intraperitoneal inoculation of encapsulated SFV-LacZ particles in SCID mice implanted with LNCaP prostate tumors. Furthermore, intravenous administration of encapsulated SFV particles expressing IL-12 in melanoma and kidney carcinoma patients showed 5-10 fold increase in IL-12 secretion and a high safety profile [26,106].

An interesting approach has been to apply miRNAs to influence targeted replication. In this context, engineering of SFV vectors with the neuron-specific miR124 reduced virus spread within the central nervous system in BALB/c mice [88]. Additionally, the presence of miR124 resulted in tolerance to IFN-1 and enhanced oncolytic activity.

## 6. Conclusions

In summary, alphavirus vectors have demonstrated promising potential in cancer immunotherapy. Clearly, the easy and rapid production of high titer recombinant particles and the possibility to administer in vitro transcribed RNA or layered plasmid DNA vectors are great assets. Moreover, the transient nature of high level recombinant protein expression and rapid RNA replication with no risk of genome integration, are additional features making alphavirus vectors attractive. In relation to other viral vectors, 100–1000-fold lower doses of SFV were needed to elicit therapeutic efficacy of HPV E6-E7 immunization in comparison to vaccination with adenovirus vectors [107]. The tumor-targeting property of oncolytic alphaviruses has offered enhanced tumor growth inhibition and tumor eradication. Several studies have also demonstrated prolonged survival of animals subjected to immunizations. A number of approaches for improved therapeutic efficacy has been evaluated. These include combination treatment with, for instance, VEGF to increase the leakiness of tumor vasculature, co-infection of alphavirus and VV vectors and introduction of miRNA sequences in the vector. The drawback of using alphaviruses in cancer immunotherapy, however, relates to the lack of efficient packaging cell lines and systems for the production of high titer virus, especially of good manufacturing practice (GMP) grade. Although successful production of SFV, SIN [108] and XJ-160 [109] has been achieved, the titers are insufficient for large-scale production. Another concern has been the broad host range, which supports the need to develop vectors capable of targeting tumor cells. That is obviously why application of oncolytic alphaviruses is an attractive alternative. An important issue related to the application of alphaviruses for immunotherapy is the potential of vector-specific immune responses. Generally, alphaviruses are described to possess no widespread immunity in human and animal populations, although some epidemics related to SFV, SIN, VEE and CHIK have been documented [110]. Furthermore, immunization studies with alphaviruses have indicated that alphavirus vectors, especially replication-deficient vectors, generate only low immunogenicity, allowing repeated vector administration. Moreover, use of RNA replicons further reduces the vector immunogenicity.

One shortcoming of alphaviruses in relation to several other viral vectors is the modest number of clinical trials conducted or planned. Actually, it is not that surprising as according to PubMed there are ten times more published studies in the area of cancer immunotherapy for adenoviruses than alphaviruses (www.ncbi.nlm.nih.gov/pubmed/). However, although no major breakthrough has been achieved so far, the conducted studies confirmed safe administration of alphaviruses. The next steps will therefore be to optimize vector constructs and dose administration to improve prophylactic and therapeutic efficacy of oncolytic alphaviruses.

## Figures and Tables

**Table 1:** Examples of oncolytic alphavirus vectors applied for cancer immunotherapy.

Cancer	Vector/Gene	Effect	Reference
Blood	SIN	tumor targeting, prolonged survival	[80]
Bone	SFV/*EGFP*	tumor regression, improved survival	[75]
	SFV/*EGFP*	tumor cell killing	[79]
Brain and neuronal	SFV/*IL-12*	tumor regression, improved survival	[77]
	SFV/*EGFP*	tumor eradication	[71]
	SFV4-miRT124	tumor regression, improved survival	[96]
	SIN/*GFP*	tumor killing in vivo	[72]
	CHIK	apoptosis in U87MG cells	[73]
	SIN AR339	tumor regression in vivo	[68]
Cervix	SIN AR339	suppression of ascites formation	[65]
Colon	SFV/*EGFP*	tumor regression	[79]
	SFV/*IL12* + PD1	tumor regression, prolonged survival	[82]
Kidney	encSFV/*IL12*	enhanced IL-12 secretion in patients	[105]
Liver	M1	tumor growth inhibition	[83]
Lung	SFV/*EGFP*	tumor regression	[75]
Melanoma	SFV/*EGFP*	tumor regression in mouse, rat	[71]
	SFV/*EGFP*	tumor regression in mice	[70]
	SFV/*IL12* + PD1	tumor regression, prolonged survival	[82]
	encSFV/*IL12*	enhanced IL-12 secretion in patients	[105]
Oral	SIN AR339	apoptosis in OSCC cell lines	[67]
Ovarian	SIN AR339	suppression of ascites formation	[65]
	SFV + VV/*OVA*	tumor killing	[81]

CHIK: Chikungunya virus; EGFP: enhanced green fluorescent protein; encSFV: liposome encapsulated SFV; IL-12: interleukin 12; OVA: ovalbumin; OSCC: oral squamous cell carcinoma; PD1: anti-PD1 monoclonal antibody; SFV: Semliki Forest virus; SIN: Sindbis virus; VV: vaccinia virus.
